# Supplementary material for: Identification of Copy Number Variations in Four Horse Breed Populations in South Korea
Source: Animals (Basel). 2022 Dec 12;12(24):3501. doi: 10.3390/ani12243501 (PMC9774267; doi:10.3390/ani12243501)
Supplement: Supplementary file 1 [file animals-12-03501-s001.zip › Supplementary Figure.pdf]

(a)

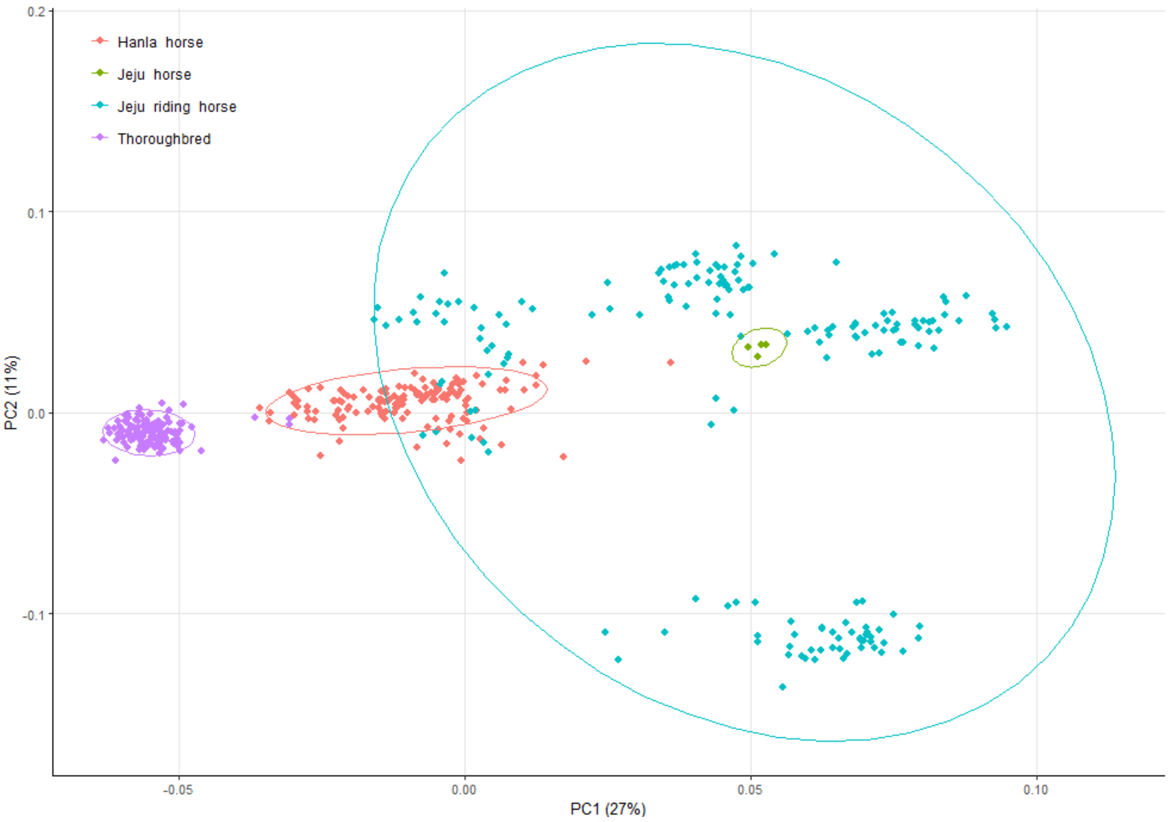

Hanla horse
Jeju horse
Jeju riding horse
Thoroughbred
0.2
0.1
0.0
-0.1
PC2 (11%)
-0.05
0.00
0.05
0.10
PC1 (27%)

(b)

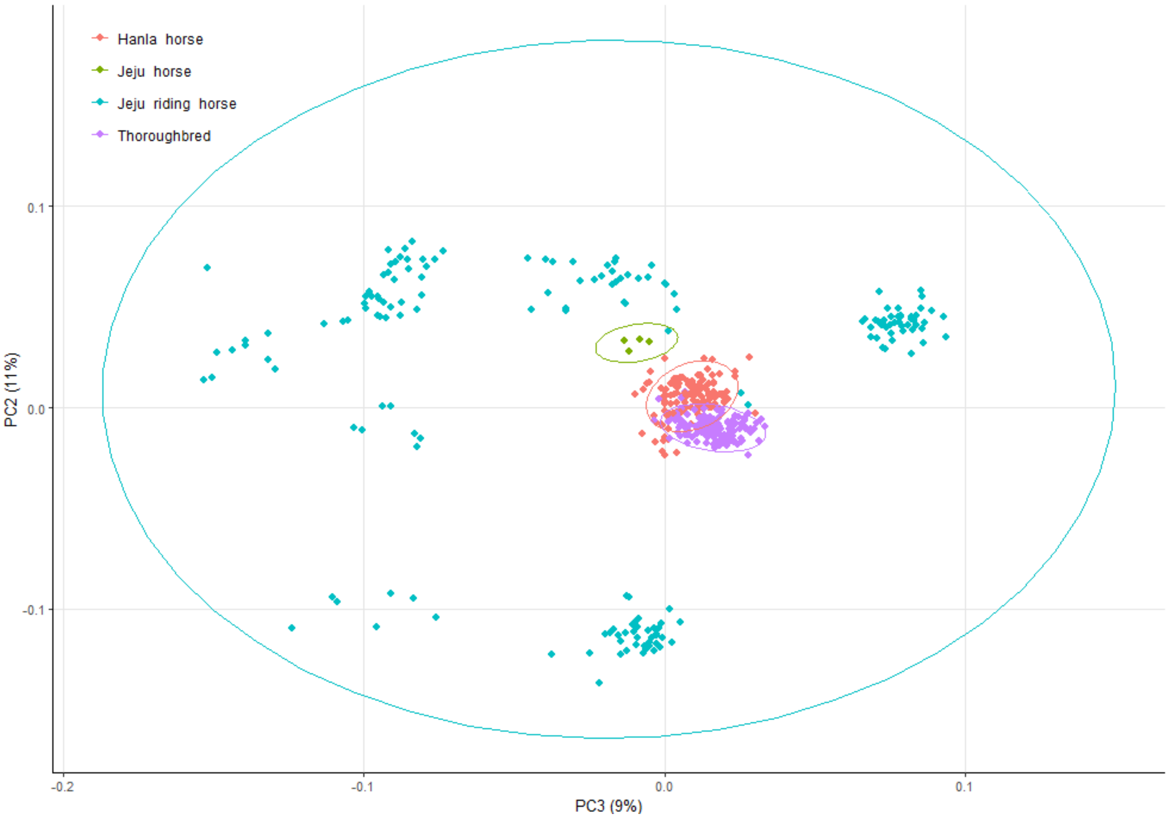

Hanla horse
Jeju horse
Jeju riding horse
Thoroughbred
0.1
0.0
-0.1
PC2 (11%)
-0.2
-0.1
0.0
0.1
PC3 (9%)

(c)

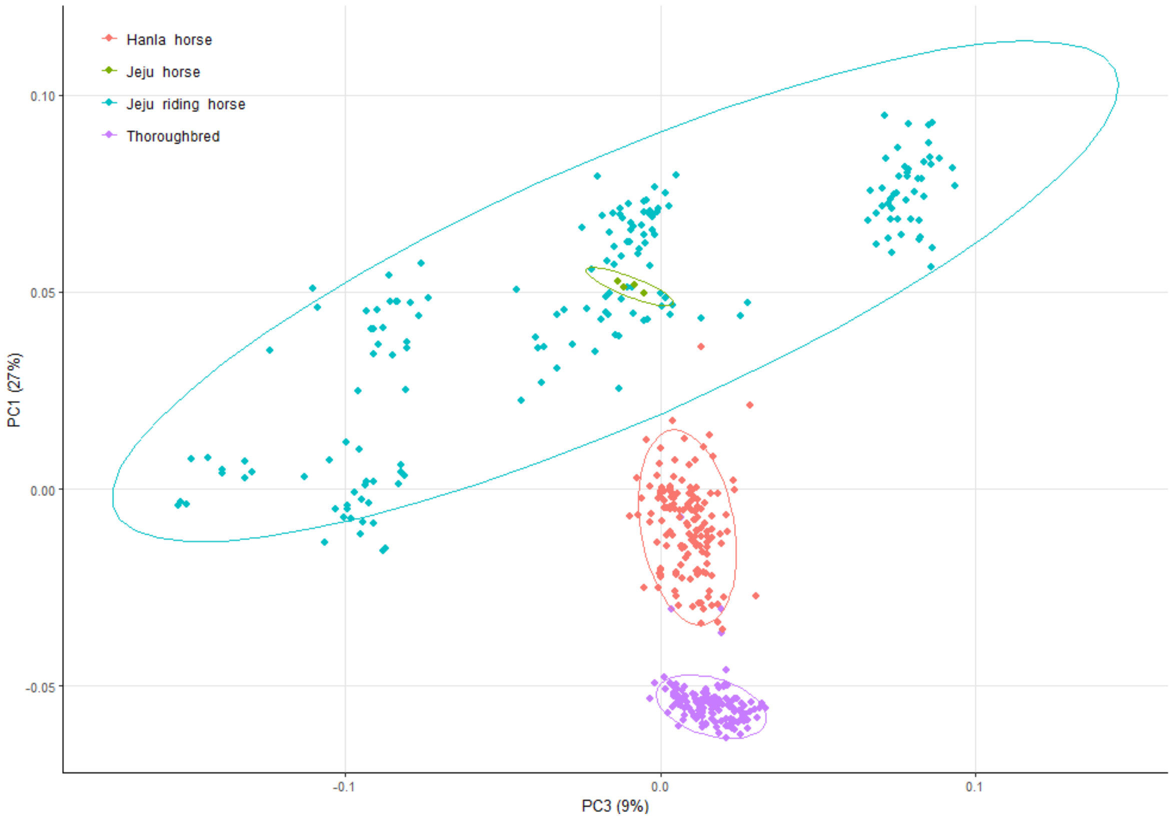

(d)

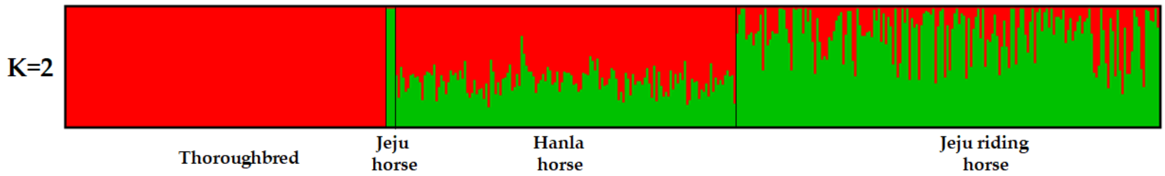

Figure S1. PCA and admixture analysis results of four horse breeds. (**a**) PCA analysis of PC1 vs. PC2; (**b**) PCA analysis of PC3 vs. PC2; (**c**) PCA analysis of PC3 vs. PC1; (**d**) Admixture analysis (K = 2)

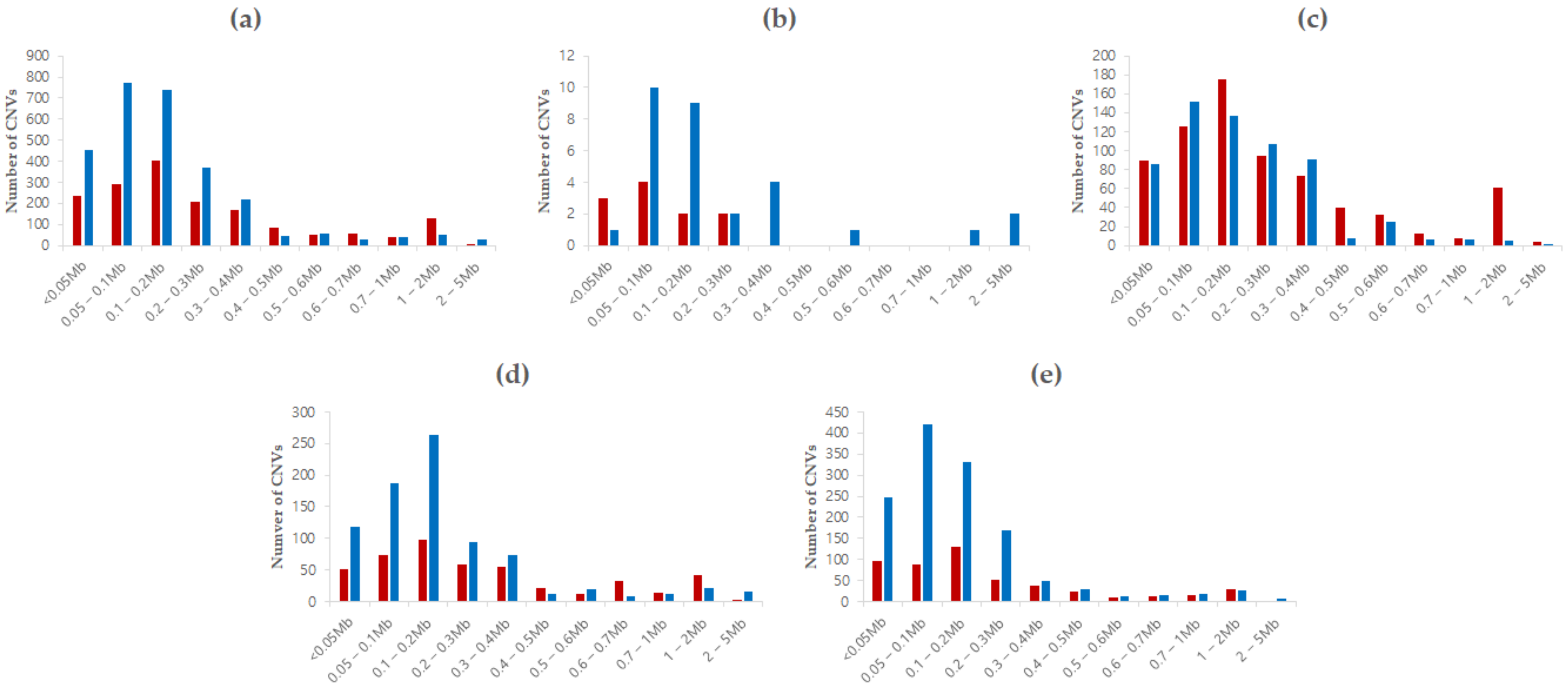

Figure S2. Gain and loss distribution by length of copy number variation (CNV) in four horse breeds in South Korea. (**a**) Total CNV distribution; (**b**) Jeju horse CNVR distribution; (**c**) Thoroughbred CNVR distribution; (**d**) Jeju riding horse CNVR distribution; (**e**) Hanla horse CNVR distribution. Red and blue represent losses and gains respectively.
